# The Story of the Insane from Year to Year

**Published:** 1904-09-10

**Authors:** 


### THE STORY OF THE INSANE FROM YEAR TO YEAR.

Sixteenth Annual Series.

### HEREFORD COUNTY ASYLUM.

This asylum contained 420 patients on the first of January, and 496 on the last day of December. There were 109 first admissions, 52 readmissions, 29 recoveries, 12 discharged relieved, and 38 deaths. The total number under treatment during the year was 581, and the average daily number resident was 453. Calculated on the admissions, the recoveries stand at 34·1 per cent, and, calculated on the average number resident, the deaths stand at 8 3 per cent. Both of these percentages are under the County Asylum average. Of the year's deaths 12 were caused by cerebral and spinal diseases, and 2 by consumption. Of the admissions, 9 owed their insanity to moral causes, 13 to intemperance in drink, and 19 to hereditary influence. Dr. Morrison again comments on the unfavourable type of the year's admissions as regards chances of recovery, and he points out that "parochial officials send to asylums persons of unsound mind, especially those advanced in years whose bodily infirmities or diseases more than their mental condition require asylum care." This is probably true of every asylum in England, and no attempt—certainly no united attempt—is made on the part of the County Council Committees to check this influx of chronic patients, the vast majority of whom could be more economically treated elsewhere; neither does Dr. Morrison tell us what he thinks should be done in this matter. Another point which deserves particular attention is the frequent changes taking place among the junior members of the staffs of asylums. Many are the allusions to this fact in our county asylum reports, and cures for the malady are not readily forthcoming. Dr. Morrison says that the "Poor-law service in which superannuation is assured appears to offer better prospects." This may be one cause; but it is not a potent one, for it seems to us unlikely that an attendant or nurse would leave asylum service where fair hopes of a moderate pension at the age of 50 exist, for a smaller if more certain pension at the age of 60. Nevertheless we look upon the settlement of the asylum pensions question as an extremely important one, and one that will not brook delay much longer. An appendix to the medical superintendent's report deals with the increasing cost of the maintenance of patients in the Hereford and, incidentally, in other asylums. It shows once again that the lowest maintenance rate exists in asylums containing about 1,000 patients; and, in spite of this and of numerous other drawbacks to large asylums, additions are still allowed to be made to asylums containing 2,000 beds, or even more.

THE ROYAL ASYLUM, PERTH.

On the 1st of January this asylum contained 129 patients; and on the 31st December there were 132 in residence. There were 42 first admissions, 7 readmissions, 7 discharged recovered, 20 discharged relieved, 10 discharged not improved, and 9 died. The total number under care during the year was 176, and the average daily number resident was 131. Calculated on the admissions, the recoveries were 21·42 per cent., and the deaths were 6·82 per cent. of the average number resident. The recoveries are considerably lower than the average at the Perth Asylum, and the deaths are about 1 per cent. higher. Three of the 9 deaths were caused by consumption. Of course the total number of deaths is so small that to say 33⅓ per cent. of the deaths were owing to phthisis, although true, might convey quite an erroneous idea of the prevalence of that disease in the asylum. But Dr. Urquhart has had two shelters erected for the treatment of consumptives. These are in occupation, and his experience has been favourable—so much so, that he thinks it points to an extension of open-air treatment to other patients than consumptives. The patients resident in the Perth Asylum at the close of the year were most unfavourably placed as regards chances of recovery, and Dr. Urquhart does not believe that more than 12 of the total 132 will recover; and we can understand this estimate when we learn that 6 were epileptic, 2 were general paralytics, 22 were over 60 years of age, and 14 were over 70. Many were suicidal and 15 were dangerous cases. It is well known that Dr. Urquhart's patients enjoy much freedom, and we learn from this report that 47 were entrusted with liberty on parole and that 21 were allowed to walk beyond the grounds unattended. The average cost per head per annum was £89. The ordinary minimum rate of board for patients from districts beyond Perthshire is £84, but during last year not fewer than 40 patients from the county were admitted at rates varying from £20 to £52, a fact which shows that the governors do not neglect the charitable side of their duties.

STIRLING DISTRICT ASYLUM.

At the beginning of the year the numbers were 683 in residence, and at the end of the year 684. There were 221 first admissions, 53 readmissions, 110 recoveries, 64 discharged relieved, and 98 deaths. The average number resident was 682, and the total number under treatment was 957. The recoveries stand at 40 1 per cent. of the admissions, and the deaths at 10·2 per cent. of the average number resident. Thirty-six of the deaths were caused by cerebral and spinal diseases, 15 by consumption, 9 by pneumonia, and 2 by enteritis. Of the year's admissions only 8 were due to moral causes, but 53 were due to intemperance in drink, 45 to hereditary influence and 9 were unknown. Dr. Robertson's report is a very interesting one, and the points concerning the increase of insanity, and those relating to the employment of nurses in the male wards are very ably put. There would seem to be a somewhat lower proportion of insanity in Stirling than in other districts of Scotland, and Dr. Robertson points out that this is probably due not to the possession of superior qualities of brain, but rather to the fact there is a higher proportion of juveniles and a higher death-rate among the children. The amount of insanity is, however, increasing in Stirling. We presume Dr. Robertson means that the number of certified lunatics is increasing, and he states very clearly the causes for this fact:—The conception of insanity includes more than it did; the medical idea of the disease has taken firmer root; confidence in the management of asylums; and the development in the mind of the general practitioner as regards the proper treatment of insanity. Undoubtedly all these causes have been and still are in operation. As regards the employment of nurses in the male wards, Dr. Robertson asks the question: Do the nurses care for the insane men under their charge as well or better than the attendants? And he has no hesitation in answering that "with the limitations and precautions adopted here they do it better." The Colney Hatch fire alarmed Dr. Robertson, as well as a good many other asylum officials, and he gave instructions that the doors of the hospital block were not to be check-locked by night. All the nurses disliked this innovation, as it made them nervous; but we are glad to learn that these nervous fears have vanished. Asylum traditions are like other traditions, and die very hard. Locked doors by day are still the rule in English county asylums, and in many of them prison-like formalities have to be gone through before even the outer gates can be passed. There are very nearly as many conservative physicians in the present day as in Dr. Conolly's time, but Dr. Robertson should have known that Conolly was not the first, nor if we remember rightly, even the second, who adopted non-restraint in England. If the employment of nurses in male wards be really a reform, it will go on, slowly it may be, and Dr. Robertson may console himself with the reflection that truth has to creep in very stealthily like a thief in the night, wherever an old tradition is in the doorway. The committee say that Dr. Robertson has "instituted reforms of a progressive character which have attracted great interest, and which have been attended by undoubted success."

